# Genotype is an important determinant factor of host susceptibility to periodontitis in the Collaborative Cross and inbred mouse populations

**DOI:** 10.1186/1471-2156-14-68

**Published:** 2013-08-09

**Authors:** Ariel Shusterman, Yasser Salyma, Aysar Nashef, Morris Soller, Asaf Wilensky, Richard Mott, Ervin I Weiss, Yael Houri-Haddad, Fuad A Iraqi

**Affiliations:** 1 Department of Prosthodontics, Faculty of Dental Medicine, Hadassah Medical Centers and The Hebrew University, Jerusalem, Israel; 2 Department of Clinical Microbiology and Immunology, Sackler Faculty of Medicine, Tel Aviv University, Tel Aviv, Israel; 3 Department of Genetics, Hebrew University, Jerusalem, Israel; 4 Department of Periodontology, Faculty of Dental Medicine, Hadassah Medical Centers and The Hebrew University, Jerusalem, Israel; 5 Wellcome Trust Human Genome Centre, Oxford University, Oxford OX3 7BN, UK

**Keywords:** Periodontal infection, Experimental periodontitis, microCT, Collaborative cross, Genes, Heritability

## Abstract

**Background:**

Periodontal infection (Periodontitis) is a chronic inflammatory disease, which results in the breakdown of the supporting tissues of the teeth. Previous epidemiological studies have suggested that resistance to chronic periodontitis is controlled to some extent by genetic factors of the host. The aim of this study was to determine the phenotypic response of inbred and Collaborative Cross (CC) mouse populations to periodontal bacterial challenge, using an experimental periodontitis model. In this model, mice are co-infected with *Porphyromonas gingivalis* and *Fusobacterium nucleatum*, bacterial strains associated with human periodontal disease. Six weeks following the infection, the maxillary jaws were harvested and analyzed for alveolar bone loss relative to uninfected controls, using computerized microtomography (microCT). Initially, four commercial inbred mouse strains were examined to calibrate the procedure and test for gender effects. Subsequently, we applied the same protocol to 23 lines (at inbreeding generations 10–18) from the newly developed mouse genetic reference population, the Collaborative Cross (CC) to determine heritability and genetic variation of control bone volume prior to infection (CBV, naïve bone volume around the teeth of uninfected mice), and residual bone volume (RBV, bone volume after infection) and loss of bone volume (LBV, the difference between CBV and RBV) following infection.

**Results:**

BALB/CJ mice were highly susceptible (P<0.05) whereas DBA/2J, C57BL/6J and A/J mice were resistant. Six lines of the tested CC population were susceptible, whereas the remaining lines were resistant to alveolar bone loss. Gender effects on bone volume were tested across the four inbred and 23 CC lines, and found not to be significant. Based on ANOVA analyses, broad-sense heritabilities were statistically significant and equal to 0.4 for CBV and 0.2 for LBV.

**Conclusions:**

The moderate heritability values indicate that the variation in host susceptibility to the disease is controlled to an appreciable extent by genetic factors. These results strongly support the possibility of using the Collaborative Cross, as well as developing dedicated F2 (resistant x susceptible inbred strains) resource populations, for future dissection of genetic factors in periodontitis.

## Background

Periodontal infection is the most common chronic inflammatory disease in humans, resulting in formation of periodontal pockets, leading to destruction of tooth-supporting tissues and alveolar bone resorption and culminating in tooth loss [1]. The disease is initiated by periodontal pathogenic bacteria which accumulate as subgingival biofilms in periodontal pockets [2], and/or by bacterial by-products such as lipopolysaccharide (LPS) [3]. Recently, several lines of evidence suggest that there is a significant genetic component associated with the resistance to chronic periodontitis [4-6] and candidate gene analysis has shown association of resistance with genetic polymorphism at genes involved in the immune response [7,8].

The flora found in chronic periodontitis is a mixture of many bacterial species. Although *Poryphyromonas gingivalis* and *Fusobacterium nucleatum,* have been strongly implicated in periodontitis development, several other bacterial species have been found in individuals with the disease [9-12], but no single species, including *P. gingivalis* and *F. nucleatum,* is present in all periodontal patients [13]. The bacterial species found in periodontal patients are also present in healthy subjects [10,12]. Although clinical and epidemiological studies have provided strong evidence for a genetic predisposition to chronic periodontal disease [4], it would be very costly and logistically complex to conduct a genome-wide search for genetic factors associated with the infection in human populations, primarily because of the difficulty in controlling the challenge and obtaining a sufficiently large sample size. Thus, despite major advances in the awareness of genetic risk factors for periodontal disease, the specific loci involved in susceptibility to periodontal disease remain unknown [6].

Inbred mouse strains often show differences in genetic predisposition to infectious diseases. In such cases, the mode of inheritance, whether mono - or polygenic, can be elucidated by genetic analysis [14,15]. Such approaches have been used successfully to identify murine loci conferring resistance to bacterial, viral and parasitic diseases [16-20]. Once genes conferring resistance to bacterial pathogens have been identified in a mouse model, genetic analysis and cloning of the orthologous genes can be extended to humans [21].

A number of mouse resource populations have been proposed for genetic dissection of complex traits. Among these is the Collaborative Cross (CC), designed specifically for complex trait analysis. This unique genetic resource, proposed as a community effort of the complex trait consortium (CTC, http://www.complextrait.org) will eventually comprise a set of ~ 500 Recombinant Inbred Lines (RIL) that are being created by full reciprocal 8-way matings of 8 different mouse strains [22-25].

In this study, we used the oral mixed-infection system of the two anaerobic Gram- negative bacteria *P. gingivalis* and *F. nucleatum* to induce experimental periodontitis in mice, and the computerized microtomography (microCT) technique to assess residual alveolar bone volume after infection [26]. Initially we challenged four standard laboratory strains to examine gender and genetic effects on susceptibility to periodontal disease. Following this we challenged 23 lines of the CC population (out of a total of 120 CC lines now under development in our laboratory), to test the potential usefulness of this resource population for the genetic dissection of periodontal disease. Our results show absence of gender effects, but a significant variation in the control bone volume (CBV), and in the loss of bone volume (LBV) upon infection, among the four commercial inbred lines and among the CC lines. This provides a promising basis for more extensive mapping in the CC resource of murine Quantitative Trait Loci (QTL) affecting alveolar bone loss due to periodontitis.

## Results

### Gender effects

Table 1 compares the gender effects for the four commercial inbred lines and the three randomly chosen CC lines under control (i.e., uninfected) and infected conditions. Significant gender effects in CBV and RBV were not found for any of the individual lines (P= 0.8 and 0.2, respectively). Also, the overall comparison, summed across all the lines and treatments, did not show any evidence of gender effects, nor did two-way ANOVA (gender x line) for all the 23 CC lines (data not shown). Consequently, in the subsequent analyses both genders were pooled and treated as samples from the same population. Two-way ANOVA across all the CC lines showed that the gender effect on LBV was not significant (p<0.98).

**Table 1:** Test for gender differences

**Line**	**Trait**	**Male**			**Female**			
		**No.**	Mean*	**SE**	**No.**	Mean*	**SE**	**p-value**
BALB/cJ	CBV	5	6.56	0.60	5	5.98	0.21	0.39
	RBV	4	4.37	0.28	5	4.68	0.33	0.54
	LBV		2.19	0.66		1.30	0.39	
C57BL/6J	CBV	5	5.50	0.82	4	3.75	0.22	0.11
	RBV	3	5.10	0.50	4	4.60	0.44	0.49
	LBV		0.40	0.96		−0.85	0.49	
A/J	CBV	5	7.32	0.49	5	6.96	0.61	0.66
	RBV	5	7.20	0.42	5	7.68	0.43	0.45
	LBV		0.12	0.65		0.72	0.75	
DBA/2J	CBV	4	6.52	0.25	3	7.53	0.88	0.26
	RBV	5	6.64	0.25	4	6.95	0.43	0.52
	LBV		−0.12	0.35		0.58	0.98	
CC034/TAU	CBV	8	3.35	0.45	2	3.85	0.75	0.62
	RBV	5	3.62	0.39	2	5.05	0.95	0.14
	LBV		−0.27	0.60		−0.12	1.21	
CC188/TAU	CBV	2	6.35	0.15	3	4.83	0.44	0.08
	RBV	2	5.15	0.05	3	5.80	0.71	0.53
	LBV		1.20	0.16		−0.97	0.84	
CC711/TAU	CBV	6	6.52	0.62	7	6.10	0.64	0.65
	RBV	4	4.93	0.69	6	4.72	0.85	0.87
	LBV		1.59	0.93		1.38	1.06	

Comparison of control bone volume before infection (CBV), and residual bone volume (RBV) and loss of bone volume (LBV) following infection in males and females. Results are shown for four standard laboratory pure lines, and for three randomly chosen TAU CC lines.

*CBV* Control Bone Volume.

*RBV* Residual Bone Volume.

*No.* number of mice.

*SE* standard error.

* = × 10^-3^mm^3^.

### Consistency of infection

**Figure 1:**
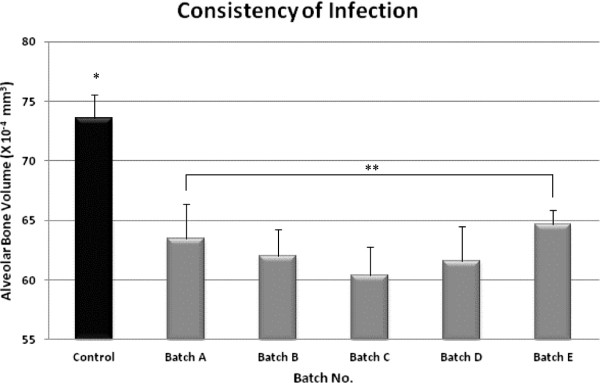
**Residual alveolar bone volume (RBV) following infection (RBV) in BALB/c mice across five independent trials (A through E, gray bars) compared to control bone volume (CBV) before infection, in Trial 1 (black bar).** Differences among trials, not including control, NS (P=0.57). Differences among trials including control, highly significant (P=0.004).

Figure 1 shows the consistency of infection across the five batches Using one-way ANOVA, the differences among the infected replicates were not significant (P=.569); whereas the differences among all the groups, including the control, were highly significant (P=0.004). Thus, our protocol shows consistent results. Although we did not test routinely for infection *per se*, Backer et al. [27] using the same protocol and colony-forming units, reported that *P. gingivalis* could be isolated from infected mice up to 42 days post infection. In our laboratory, we also have identified both *P. gingivalis* and *F. nucleatum* up to 6 weeks post infection using PCR (unpublished data).

### CBV, RBV and LBV for the pure lines and the CC lines

Tables 2 and 3 shows the CBV, RBV and LBV of the pure lines and the CC lines. As the four pure lines and the 23 CC lines were produced in different locations, any differences between the two groups can be attributed to this factor. Hence, their results are presented and discussed separately. Comparison of the pure lines with one another (Table 2), shows that the mean CBV of the C57BL/6J inbred line was clearly lower than that of the A/J, DBA/2J and BALB/cJ lines. The difference was significant for A/J and DBA/2J and nearly significant for BALB/cJ. Similarly, the mean RBV after infection was significantly lower for the BALB/cJ and C57BL/6J mice than for the A/J and DBA/2J mice. A comparison of the mean bone volume of the control and infected mice within the lines provides an estimate of LBV due to infection (In this comparison, BALB/cJ presented a highly significant decrease in the bone volume of the infected vs the control mice (P<0.001). The other three lines showed very little difference in mean bone volume (whether positive or negative) between the control and infected groups. Thus, the BALB/cJ mice can be classed as “susceptible”, and the other three lines as “resistant.”

**Table 2:** Characteristics of four standard laboratory pure lines with respect to control bone volume before infection (CBV), and residual bone volume (RBV) and loss of bone volume (LBV) following infection

**Line**	**CBV**		**RBV**		**LBV**	
	**No.**	Mean*	**No.**	Mean*	**No.**	Mean*
BALB/cJ	12	6.27^a,b^	9	4.54^a^	9	1.73^b^**
C57Bl/6J	9	4.72^a^	7	4.81^a^	7	−0.09^a,b^
A/J	10	7.14^b^	10	7.44^b^	10	−0.30^a^
DBA/2J	7	6.95^b^	9	6.78^b^	7	0.17^a,b^

*CBV* Control Bone Volume.

*RBV* Residual Bone Volume.

*No.* number of mice.

* = × 10^-3^mm^3^.

** = loss of bone volume following infection significant at P<0.01.

^a,b^ = Lines within a column that share the same superscript letter do not differ significantly by Duncan’s Least Significant Range test.

**Table 3:** Comparison of 23 TAU CC lines with respect to control bone volume before infection (CBV), and residual bone volume (RBV) and loss of bone volume (LBV) following infection

**Rank**	**Line**	**CBV**		**RBV**		**LBV**
		**No.**	Mean*	**No.**	Mean*	Mean*
CC01	CC026/TAU	4	10.68^d,e^	6	6.65^c,d,e^	4.03^c,d^***
CC02	CC030/TAU	8	7.05^b,c^	9	5.90^b,c,d^	1.15^a,b,c,d^
CC03	CC034/TAU	10	3.45 ^a^	4	3.45^a,b^	0^a,b^
CC04	CC057/TAU	3	11.10^d,e^	5	6.44^c,d,e^	4.66^d^***
CC05	CC072/TAU	12	6.28^a,b^	11	4.72^a,b,c^	1.56^a,b,c,d^***
CC06	CC111/TAU	8	6.34^a,b^	6	5.80^b,c,d^	0.54^a,b,c^
CC07	CC114/TAU	5	7.98^b,c^	6	10.12^e,f^	−2.14^a^
CC08	CC182/TAU	5	10.70^d,e^	5	6.02^c,d^	4.68^d^***
CC09	CC188/TAU	5	5.44 ^a^	5	5.54^b,c,d^	−0.10^a,b^
CC10	CC196/TAU	8	8.19^b,c,d^	8	6.13^c,d^	2.06^b,c,d^***
CC11	CC211/TAU	4	5.88^a,b^	6	5.50^b,c^	0.38^a,b,c^
CC12	CC219/TAU	5	5.62^a,b^	5	4.46^a,b^	1.16^a,b,c,d^
CC13	CC521/TAU	7	5.89^a,b^	5	5.50^b,c^	0.39^a,b,c^
CC14	CC530/TAU	4	5.58^a,b^	3	6.03^c,d^	−0.45^a,b^
CC15	CC534/TAU	3	6.0^a,b^	4	5.55^b,c,d^	0.45^a,b,c^
CC16	CC551/TAU	6	3.72^a^	7	2.64^a^	1.08^a,b,c,d^
CC17	CC611/TAU	4	5.83^a,b^	6	4.90^a,b,c^	0.93^a,b,c,d^
CC18	CC643/TAU	5	6.08^a,b^	3	6.07^c,d^	0.01^a,b^
CC19	CC670/TAU	6	9.93^c,d,e^	4	8.53^e,f^	1.40^a,b,c,d^
CC20	CC688/TAU	3	3.70^a^	5	4.54^a,b,c^	−0.84^a,b^
CC21	CC696/TAU	6	8.30^b,c,d,e^	4	8.20^d,e^	0.10^a,b^
CC22	CC711/TAU	13	6.29^a,b^	10	4.8^a,b,c^	1.49^a,b,c,d^**
CC23	CC785/TAU	5	6.50^a,b^	6	5.17^a,b,c^	1.33^a,b,c,d^
Mean/Total SD		139	6.80	133	5.77	1.13
			2.17		1.59	1.63

*CBV* Control Bone Volume.

*RBV* Residual Bone Volume.

*LBV* Loss of Bone volume.

*No.* number of mice.

* = × 10^-3^mm^3^.

** = P <0.01.

*** = P <0.001.

^a,b,c,d,e,f^ = Lines within a column that share the same superscript letter do not differ significantly by Duncan’s Least Significant Range test.

Table 3 shows the mean CBV, RBV and LBV for the 23 CC lines. Of these, 6 lines (CC03, 07, 09, 14, 18, 20), showed an increase in RBV following infection, resulting in a negative LBV. However, in none of the cases was the gain in bone volume significant. The remaining lines showed a decrease in RBV following infection. For 6 of the lines the loss in volume was highly significant, i.e. 25% of the CC lines were highly susceptible. The remaining lines were probably a mixture of resistant lines and moderately susceptible lines that did not pass the significance threshold due to small sample size. There were 3 lines with exceptionally high CBV (CC01, 04, 08) and the three highest LBV values. The remaining three lines with significant LBV values (CC05, 10 and 22) did not show any association with CBV, and were only about half as high the apparent association between CBV and LBV. For the CC01, 04 and 08 lines, we raised the possibility that LBV may be proportional to CBV. To check this, we calculated pLBV (LBV as a proportion of CBV) for the 23 CC lines, and examined the correlations among the various parameters with and without the three exceptional lines. As the CBV and RBV were measured in different animals, it should be noted that the correlations between the means of the lines for the three parameters represent the genetic correlation between the pairs of traits, environmental correlation is not included. The overall correlation between LBV and CBV in the CC lines was high (r=0.72). However, this was due in large part to the three exceptional lines: CC01, 04, and 08. If the three lines are excluded, the correlation drops to 0.31, and is no longer statistically significant (P=0.09), indicating that for most lines, CBV was not a major factor in determining LBV. The correlation between LBV and pLBV was very high regardless of whether all the data were analyzed (r = 0.89, P<0.001) or only the data excluding the three exceptional lines (r=0.91, P<0.001). Thus, LBV was the major factor determining pLBV. For these reasons, as noted above, the remaining analyses were based on LBV.

### Heritability

Table 4 shows ANOVA analysis and heritability calculations for the 23 CC lines. There were highly significant differences among the lines for all three parameters, CBV, RBV and LBV. The corresponding heritability estimates were 0.40, 0.40 and 0.20. The genetic variance (VarG) was greatest for CBV, almost twice that for RBV. However, error variance for CBV was also greater than for RBV in the same proportion. Consequently, the heritability was exactly the same (0.40) for both. Genetic variance was least for LBV and error variance was greatest, so that the heritability for this parameter (0.20) was only half as large. Nevertheless, there is clear genetic variance among the CC lines in resistance to periodontitis, as expressed by LBV. However, it should be realized that for CBV and RBV, the variation caused by environmental factors within the CC lines is 50% greater than that among the line variations caused by genetic factors; and for LBV it is fourfold greater. Thus, these traits are strongly affected by environmental factors and are independent of genetic variation.

**Table 4:** Heritability calculations for CBV, RBV and LBV

**Parameter**	**CBV**	**RBV**	**LBV**
MS Between*	24.09	14.08	12.21
MS within=VarE	4.58	2.82	5.21
P-value	<0.001	<0.001	0.002
No.	6.32	6.05	5.32
VarG	3.09	1.86	1.32
H^2^	0.40	0.40	0.20

*CBV* Control Bone Volume.

*RBV* Residual Bone Volume.

*LBV* Loss of Bone volume.

No. = average number of progeny/line.

* = × 10^-3^mm^3^.

d.f. Between = 22 for all treatments;

d.f. Within = 116, 110, and 94 for CBV, RBV and LBV, respectively.

MS between and MS within: Mean square between CC lines and within CC lines as obtained from one-way ANOVA.

VarE and VarG = environmental and genetic components of variance, respectively.

H^2^_=_ estimate of broad sense heritability.

### Segregation analysis

**Figure 2:**
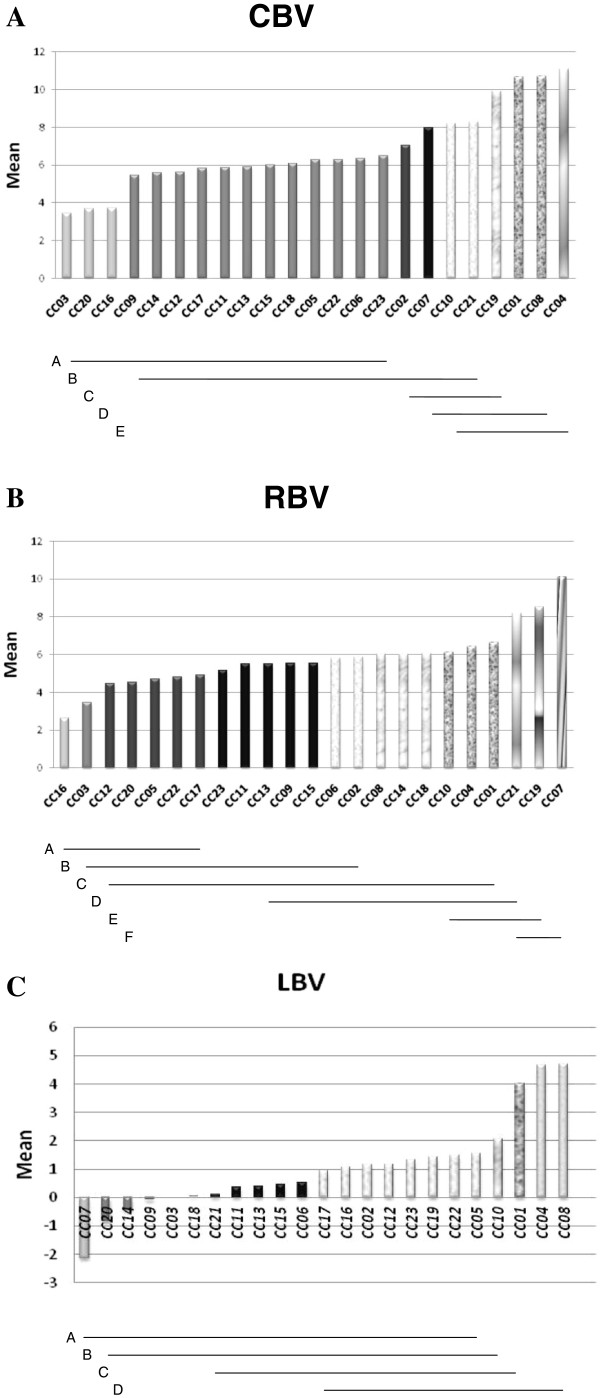
**Collaborative Cross (CC) strains arranged in increasing order of mean magnitude. A**: Control bone volume before infection (CBV); **B**: Residual bone volume following infection (RBV); **C**: Loss of bone volume following infection (LBV). Horizontal lines labeled A to E indicate groups of CC strains that carry the same superscript letter. For example, in Figure 2**A**, strain CC12 is underlined by lines **A** and **B**. This tells us that it carries superscript letters A and B; on the same rules, Strain CC19 carries superscript letters **C**, **D**, **E**, and so on for the other CC strains. Strains that carry the same superscript letters do not differ significantly from one another by Duncan’s Least Significant Range (LSR) test. Thus, Strain CC12 does not differ significantly from any of the strains ranging from CC03 on the left to CC21 on the right, but does differ from Strains CC19 and up. Strain CC19, does not differ from strains ranging from CC02 on the left to CC04 on the right, but does differ from Strains CC23 and down. CBV = Control Bone Volume; RBV = Residual Bone Volume; LBV = Loss of Bone volume. All mean values should be multiplied by ×10-3 mm3.

Figure 2A shows the mean CBV for the CC lines, arranged in increasing order of magnitude. Lines that do not share a common superscript letter are significantly different in Duncan’s Least Significant Range )LSR) Test. There was a more or less continuous distribution of values across a very large range, with an almost threefold difference in bone volume between the three lowest and the three highest lines. The LSR divides the lines into five groups, which overlap with one another to a greater or lesser extent, so that it is difficult to distinguish well separated groups. Nevertheless, there are major gaps in the distribution between ranks 3 and 4, and between ranks 19 and 20. This results in a situation where half of the lines are grouped in a narrow cluster from CBV 5.44 to 6.50. In the low tail of CBV, there is a distinct gap, and then a group of three clearly separated lines (CBV 3.45-3.72). In the high tail of CBV there is a long series of lines with higher values (CBV 7.05 to 11.10), with a strong gap separating the four highest lines. Thus, at a minimum there appear to be three distinct groups: A low group of three lines (CBV 3.45 to 3.72); an intermediate group of 16 lines (ranks 4 to 19, CBV 5.44 to 8.30); and a high group of four lines (CBV 9.93 to 11.10). Differences in CBV among lines may represent differences in the detailed anatomical structure of the maxillary bone.

The LSR analysis of RBV (Figure 2B) was quite similar to that for CBV, with a 2.5-fold difference between the lowest and highest three lines. Here, too, there were three distinct groups, one at each of the two extreme tails of the trait distribution set off by large gaps from the large intermediate group. There is a complete overlap between the CBV and RBV lines in the low tails of both traits; almost complete overlap in the high tails (4 of the 5 highest lines are the same for both traits). Across both extremes there are five lines for RBV. Finally, LSR analysis for LBV (Figure 2C) also shares many of the features of the CBV analysis. The lowest three lines actually gained bone volume following infection, and are separated from the remaining lines by a large gap. However, this was not statistically significant, as they did not differ according to LSR from the lines that lost only a small volume. Thus, it may be best to consider them as lines that were resistant to infection, retaining the possibility that they have “positive resistance,” i.e., respond to infection by actively countering bone loss. In contrast, the three extreme high loss lines do differ statistically from the intermediate group. These three lines correspond precisely to the extreme high CBV group. It is possible that the special anatomical structure that resulted in high CBV also resulted in high susceptibility to bone loss.

The very wide variation presented by the CC lines, is best interpreted as resulting from segregation of a small number of QTL of large effect. Many QTL of small effect would be expected to generate a tighter concentration of lines about the mean. The results of initial QTL mapping studies already implemented in the CC resource e.g., see [28-30] have shown that the wild subspecies included in the parental founders of the CC indeed introduced genes of large effect into the this population. The results of the present study would be consistent with these findings.

## Discussion

Our results confirm the validity and accuracy of the oral infection challenge and the application of microCT in a mouse model for studying periodontal infection (periodontitis). Furthermore, this is the first report to present results from mixed infection in commercial inbred mouse lines and in the newly developed CC mouse resource population. Our results on the commercial inbred lines under control conditions are consistent with those of Baker et al. [27], showing that CBV varies among strains of inbred mice. This was also observed in the CC mice. Although for a small number of CC lines, within-line analyses showed significant differences between males and females (data not shown); significance in each case was marginal and was lost when the results were corrected for the multiple-test nature of the analysis. The absence of significant gender effects is also supported by the results of one- and two-way ANOVA analyses. Random environmental factors other than gender do play a strong role in generating variation in CBV, RBV and LBV; within the CC lines. Within trait variation was greater than the genetic component of variation between lines. Nevertheless, the highly significant differences in trait values among lines and moderate trait heritabilities, indicate that line genotype is an important determinant of the differences in values of all three traits. These results support the possibility of future mapping of QTL and subsequent identification of host genes controlling CBV, RBV and LBV in the CC resource.

Upon comparison of the CBV of the CC lines with that of the four classical inbred strains we found that six CC lines (CC01,CC04, CC08, CC10, CC19 and CC21) significantly exceeded the highest CBV value of inbred strain A/J, whereas one line (CC16) had a ;lower CBV than the lowest inbred strain, C57BL/6J. Commonly used laboratory mouse strains, which originate in a small sample of founders; have a remarkably high level of shared ancestry, largely contributed by the Mus musculus domesticus subspecies, and show limited diversity. In contrast, wild-derived inbred strains encompass genetic variation accumulated over ~ one million years: each classical laboratory strain differs from the reference C57BL/6J by ~ 4 million single-nucleotide polymorphisms (SNPs), whereas the wild-derived strains CAST/Ej and PWK/hJ each differ by 17 million SNPs, and WSB/EiJ by 6 million [31]. These three wild-derived strains are part of the eight founders of the CC population (A/J, C57BL/6J, 129S1/SvImJ, NOD/LtJ, NZO/HiLtJ, CAST/Ei, PWK/PhJ, and WSB/EiJ [22]). Therefore, the high genetic diversity in the CC population means that phenotype could be observed and subsequently QTLs, which would not have been visible in a cross between classical strains, that involved contrasts between alleles segregating between the wild-derived strains could be mapped [28].

In three of the lines, with exceptionally high CBV, there was also exceptionally high LBV. Thus, it is possible that in some cases, CBV may have a direct or indirect effect on the development and outcome of periodontal disease.

Examination of the distribution of mean trait values for the CC lines, raised the possibility that the bulk of the variation in the target traits may be due to the segregation of a small number (2 to 4) of genes, each with appreciable effect. Currently, we are extending the study to about 100 lines of the CC mouse population, which should enable us to map QTL associated with host susceptibility to the disease at small genomic intervals, and test this possibility. In parallel to the CC study, a classical F2 approach using a BALB/cJ (susceptible) x A/J (resistant) resource population for mapping QTL associated with host susceptibility to periodontitis was established complementary to the CC study. By using both approaches we expect to achieve better convergence of QTL affecting the susceptibility to periodontal disease.

In both studies, serum is being prepared and gingiva and spleen are being harvested in order to investigate changes in gene expression between the different lines at a later stage, with a view to identifying candidate genes affecting host resistance and susceptibility to the infection. An understanding of gene expression levels and subsequent changes due to infection with periodontal pathogens could provide new directions for identifying key host molecules that confer resistance and susceptibility to this complex disease.

## Conclusions

Dissection of the complex genetics of host resistance was the thrust of our study. Our results support our principal planned approach of exploiting the oral infection in a mouse model to identify QTL, by using the CC and F2 resource populations, as important sources for identifying genetic factors affecting host susceptibility to periodontal disease. Once the genetic basis of periodontal disease susceptibility is understood, such information may be of diagnostic and therapeutic value.

## Methods

### Mouse populations

The study was based on four commercially available inbred mouse lines and 23 newly developed lines from the CC mouse resource population.

#### Commercial inbred lines

Mice aged 28 weeks (10 males and 10 females) from each of the inbred mouse strains BALB/cJ, DBA/2J, C57BL/6J and A/J were purchased from Jackson Laboratory, Bar Harbor, Maine, USA. The infection challenge of the inbred lines was carried out in the specific pathogen-free (SPF) unit of the animal facility at the Hebrew University in Jerusalem, Hadassah Hospital (HUJHH). The mice were kept on a 12-hour light/dark cycle and received distilled water and chow *ad libitum*. All experimental mice and protocols were approved by the Institutional Animal Care and Use Committee of HUJHH (approval number: MD-08-10913-3).

#### Collaborative cross lines

A total 272 7- to 8-week-old mice (103 females and 169 males) from 23 different CC mouse lines (average: 11.8 mice per line), were provided by the Small Animal Facility at Sackler Faculty of Medicine, Tel Aviv University (TAU), in which the infection challenge was carried out. The lines were at inbreeding generations F_10_-F_18_, minimum 90% homozygosity by extensive high-density genotyping. Full details of the development of these CC lines are given in Iraqi *et al.*[19]. All experimental mice and protocols were approved by the Institutional Animal Care and Use Committee of TAU (approval number: M-08-044). Mice were housed on hardwood chip bedding in open-top cages at the animal facility and were given tap water and rodent chow *ad libitum*.

### Bacteria and the oral infection challenge

*P. gingivalis* strain ATCC 381 and *F. nucleatum* strain PK 1594 were grown in peptone yeast extract containing hemin and vitamin K (Wilkins Chalgren broth, Oxoid Ltd, UK), in an anaerobic chamber with 85% N_2_, 5% H_2_ and 10% CO_2_ followed by three washes in phosphate-buffered-saline (PBS). The bacterial concentration was spectrophotometrically standardized to OD_650nm_ = 0.1 for *P. gingivalis*, corresponding to 10^10^ bacteria/ml [32], and OD_660nm_ = 0.26 for *F. nucleatum*, corresponding to 10^9^ bacteria/ml [33]. Mice from each commercial inbred line were divided into infected and control groups, 10 mice in each group, equally divided between males and females. To allow controlled infection, the normal oral flora of the mice was suppressed by treating with sulfamethoxazole/ trimethoprim (0.08% and 0.016%, respectively, in drinking water *ad libitum* for 10 days, followed by a three-day wash-out, antibiotic-free period), before applying the mixed infection with *P. gingivalis* and *F. nucleatum* (400 μl containing 10^9^ bacteria/ml of each pathogen), at days 0, 2 and 4, using 2% carboxymethycellulose in PBS [26]. Control mouse groups were treated with PBS alone. Control and challenged mice of all the lines were reared intermixed. The mice were sacrificed 42 days post infection, and their maxillae were harvested for microCT analysis.

Analysis of the commercial inbred lines and the CC lines showed no difference in RBV between males and females (see below). Therefore, for evaluation of the 23 CC lines, 139 control and 133 challenge mice (average, 6 mice/ group and line) were tested as described above, without consideration of gender.

### MicroCT technique

**Figure 3:**
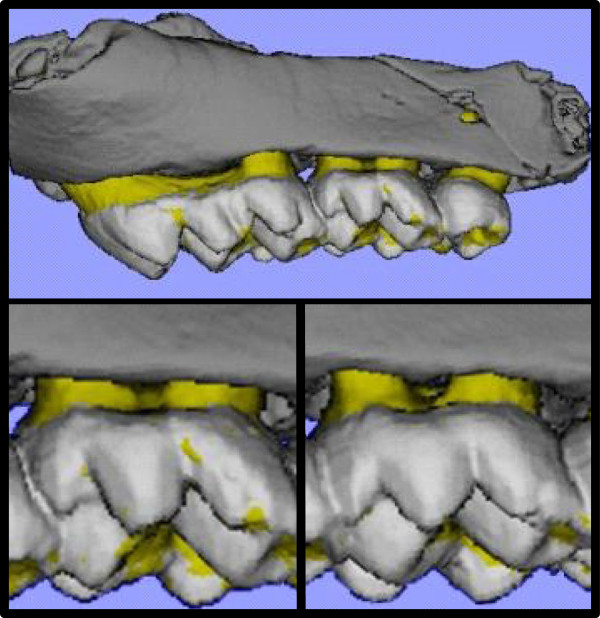
**Left mouse hemi-maxillae as reconstructed by micro- chromatography (micro-CT) of mice from line CC026/TAU of the Collaborative Cross mouse population.** Upper frame, entire uninfected left-hemi maxilla. Lower left frame, expanded view of uninfected left hemi-maxilla. Lower right frame, expanded view of post-infection left hemi-maxilla. White, enamel; yellow, dentin and cementum; gray, alveolar bone. Horizontal resorption is measured as the distance from the cemento-enamel junction (CEJ, the line between the yellow and gray colors) to the alveolar bone crest.

Maxillary hemi-jaws were analyzed by compact fan-beam-type computerized tomography (μCT 40, Scanc Medical, Bassersdorf, Switzerland). Samples were placed in a cylindrical sample holder and ~ 200 microtomographic slices with increments of 12 μm were obtained, covering the entire bucco-palatal width of each hemi-jaw. Figure 3 shows hemi-maxillae, as reconstructed by microCT, of uninfected (upper, lower left) and infected (lower right) groups of mice tested in this experiment. We found that bone volume loss due to infection occurred in a horizontal zone across the tooth bearing area in the maxilla, but did not penetrate deeply in a vertical direction from this horizontal zone. Consequently, for calculation of bone volume in control and challenged individuals, a reference line was set throughout the microtomographic slices at a set distance from the cemento-enamel junction, chosen to be below the horizontal zone of destruction. The results are presented as the coronal residual bone volume (RBV) above the reference line in mm^3^[34].

To check consistency of infection, we compared RBV after induced oral mixed infection in five independent replicates using the above protocol. The different replicates were tested at different times, including a new cycle of bacterial growth and harvesting for each replicate. We used 6 or 7 BALB/cJ female mice (5-6-weeks of age) in each round. In addition, there was a single control group of 7 females infected with PBS. RBV was measured as described above. To test for consistency of results, one-way ANOVA of RBV after challenge was carried out between the different replicates, including, and not including the control group (Figure 1).

### Data analysis

Data analysis was performed using the statistical software packages SigmaStat, (Jandel Scientific, San Rafael, CA, USA) and SPSS version 17 (SPSS Inc, Chicago, USA). To determine whether gender and line affected the periodontitis results, two-way ANOVA with CC line and gender as main effects was carried out separately on the bone volume data from the control groups, and from the infected groups.

### Measure of susceptibility to periodontal bone loss

Bone volume was calculated relative to the reference line. Consequently, bone volume depended on the distance of the reference line from the coronal alveolar bone line. This can be expected to vary among mice within a line and among lines, according to the specific anatomical structure of the jaw. Therefore, CBV and RBV can vary among lines due to this factor alone, and this will generate a strong correlation between CBV and RBV. However, since the reference line for calculation of bone volume was set to be below the zone of infection, all bone loss following the induced periodontal disease occurs within the measured area. Thus, all changes in bone volume following the induced periodontal disease are included in LBV.

The correlation between CBV and LBV across all CC lines was high, suggesting that CBV was a factor in LBV. However, this was almost completely due to three exceptional lines, which had both a very high CBV and a very high LBV. When these were excluded from the analysis, the correlation between CBV and LBV was greatly reduced and non-significant. Taking all factors into consideration, including the statistical problematics of working with ratios, we believe that the absolute LBV will be a direct measure of susceptibility, whereas the relative bone loss as a proportion of CBV (pLBV) will also include additional “noise” variation due to disparities in the location of the reference line relative to the coronal alveolar bone line. In any event, the correlation between LBV and pLBV was very high and highly significant (see Results Section). We therefore used LBV following infection as our measure of susceptibility to periodontal bone loss.

### Estimation of heritability

#### CBV and RBV

One-way ANOVA by CC line was implemented separately for CBV and RBV. Based on these analyses, broad sense heritability (H^2^, including epistatic but not dominance effects) was calculated across the CC lines under control and challenge conditions, as follows:

*H*^2^ = *V*_*g*_/(*V*_*g*_ + *V*_*e*_)

where,

*V*_*e*_ is the environment variance within lines = MS_within_

*V*_*g*_ is the genetic variance among CC lines = (MS_between_ – V_e_)/n

n = average number of mice per line

MS = Mean Squares

The significance among the line difference in the ANOVA was taken to indicate the presence of significant differences among the CC lines in the analyzed parameter.

### LBV

As bone volume measurement is a destructive procedure, CBV and RBV cannot be measured in the same animal. Consequently, it is not possible to obtain estimates of LBV for individual animals. However, since the CC mice are at an advanced inbred stage, we can assume that genetic differences are not present among the mice in a given line. Therefore, to evaluate LBV for individual animals, the control and infected mice in each line were paired at random, and the difference between the bone volume of the CBV and RBV mice in each pair was taken as a measure of the individual LBV, as if measured in the same individual. These differences were then analyzed by ANOVA, as in the preceding section, to provide an estimate of the heritability of LBV.

### Estimation of the significance of challenge effects within individual CC lines

Examination of the SD for LBV within lines did not reveal any relationship between the mean LBV for a line and the SD of the LBV within the line. Therefore, an overall estimate of LBV error variance was obtained from the within line value of the ANOVA for this trait. Based on this value, the bone loss of the individual lines was tested for significance by the z-test

where

z = LBV/SE(LBV),

SE(LBV) = SD(LBV)/n_i_, where n_i_ is the number of pairs on which the LBV value of the i^th^ CC line is based. As SD(LBV) is based on a large number of degree of freedoms (d.f.), the z=test can be used instead of the t-test.

Duncan’s Least Significant Range Test (LSR) (Walpole and Myers, 1978) was used to group the CC lines for individual traits, with a view to inferring the underlying genetic architecture of the traits. This test includes an adjustment for the number of populations compared.

## Abbreviations

CBV: Control bone volume; CC: Collaborative cross; CTC: Complex trait consortium; RBV: Residual bone volume; LBV: Loss of bone volume; microCT: Computerized microtomography; QTL: Quantitative trait loci; VarG: The genetic variance; LSR: The Least Significant Range Test; SPF: Specific pathogen-free; HUJHH: Hebrew University in Jerusalem, Hadassah Hospital; TAU: Tel Aviv University; PBS: Phosphate-buffered-saline; pLBV: The relative bone loss as a proportion of CBV.

## Competing interests

The authors declare no potential conflict of interest with respect to financial or Non-financial competing interests, the authorship and/or publication of this article.

## Authors’ contributions

AS participated in the design of the study, carried out the mice infection, participated in data analysis and wrote first draft of the manuscript. YS and AN participated in the mice infection. MS participated in data analysis and revised and edited the first draft through the final submitted version. AW carried out the mice infection and analysis of the preliminary results (inbred lines). RM participated in the data analyses. EW and YH participated in designing the study. FI participated in designing the study and in developing the first draft of the manuscript. All authors read and approved the final manuscript. YH and FI have equal contributions.
